# Lessons from a French collaborative case–control study in cystic fibrosis patients during the 2009 A/H1N1 influenza pandemy

**DOI:** 10.1186/s12879-016-1352-2

**Published:** 2016-02-01

**Authors:** Julie Bucher, Pierre-Yves Boelle, Dominique Hubert, Muriel Lebourgeois, Nathalie Stremler, Isabelle Durieu, François Bremont, Eric Deneuville, Bertrand Delaisi, Harriet Corvol, Laurence Bassinet, Dominique Grenet, Natacha Remus, Marie Véronique Vodoff, Véronique Boussaud, Françoise Troussier, Marianne Leruez-Ville, Jean-Marc Treluyer, Odile Launay, Isabelle Sermet-Gaudelus

**Affiliations:** 1 Hôpital Necker Enfants Malades, Paris, France; 2 Hôpital Saint Antoine, Paris, France; 3 Hôpital Cochin, Paris, France; 4 CHU de la Timone, Marseille, France; 5 Centre Hospitalier Lyon sud, Lyon, France; 6 CHU Purpan, Toulouse, France; 7 CHU Hôpital Sud, Rennes, France; 8 Hôpital Robert Debré, Paris, France; 9 Hôpital Trousseau, Paris, France; 10 Centre Hospitalier Intercommunal, Créteil, France; 11 Hôpital Foch, Paris, France; 12 Centre Hospitalier, Mulhouse, France; 13 Centre Hospitalier Georges Pompidou, Paris, France; 14 CHU, Angers, France; 15 GHU Necker-Cochin, Paris, France; 16 Service de PneumoAllergologie Pédiatrique; INSERM U 1151, Hôpital Necker, 149 rue de Sévres, 75015 Paris, France

**Keywords:** Cystic fibrosis, A/H1N1 influenza, Pandemic, Oseltamivir

## Abstract

**Background:**

Viral infections such as influenza are thought to impact respiratory parameters and to promote infection with *Pseudomonas aeruginosa* in patients with cystic fibrosis (CF). However, the real morbidity of the influenza virus in CF needs to be further investigated because previous studies were only observational.

**Methods:**

CF patients were included in a case–control study (*n* = 44 cases and *n* = 371 controls) during the 2009 pandemic A/H1N1 influenza. Cases were patients with polymerase reaction chain-confirmed influenza A/H1N1 infection. Controls did not report any influenza symptoms during the same period. Sputum colonization and lung function were monitored during 1 year after inclusion.

**Results:**

Cases were significantly younger than controls (mean(SD) 14.9 years(11) versus 20.1 years (13.2) and significantly less frequently colonized with *P. aeruginosa* (34 % versus 53 %). During influenza infection, 74 % of cases had pulmonary exacerbation, 92 % had antibiotics adapted to their usual sputum colonization and 82 % were treated with oseltamivir. Two cases required lung transplantation after A/H1N1 infection (one had not received oseltamivir and the other one had been treated late). The cases received a mean number of antibiotic treatments significantly higher during the year after the influenza infection (mean(SD) 2.8 (2.4) for cases versus 1.8(2.1) for controls; *p* = 0.002). An age-matched comparison did not demonstrate any significant modification of bronchopulmonary bacterial colonization during the year after influenza infection nor any significant change in FEV1 at months 1, 3 and 12 after A/H1N1 infection.

**Conclusions:**

Our results do not demonstrate any change in sputum colonization nor significant lung disease progression after pandemic A/H1N1 influenza.

**Trial registration:**

Clinical Trials.gov registration number: NCT01499914

## Background

Cystic fibrosis (CF) is characterized by a chronic obstructive bronchopathy with bacterial colonization and recurrent infections which progresses towards an irreversible deterioration of the respiratory function and terminal respiratory failure [1]. Chronic bronchial infection with *Pseudomonas aeruginosa* was found to be a major predictive factor of lung morbidity and patient mortality [1]. The rate of pulmonary exacerbations with this microorganism is associated with increased mortality and it is important to identify pulmonary exacerbations that may be preventable. Viral infections, including influenza, are clearly correlated with an increase in the number of pulmonary exacerbations and antibiotic courses, as suggested by the increase of CF pulmonary exacerbations during the influenza season [2–14]. Moreover, and very importantly, new bacterial colonizations predominantly occur following viral upper respiratory tract infections [13, 14]. This has been related to the fact that viral infections alter host defense equilibrium and increase mucus production [10]. This promotes bacterial overgrowth, and in turn, potentiates chronic colonization because of abnormal mucociliary clearance of CF sputum and intrinsic defect in innate immunity [14]. More specifically, the relationship between influenza virus and *P. aeruginosa* infection was suggested from studies which suggested more frequent new colonization or increased in *P. aeruginosa* related exacerbation [11, 13, 15]. However, the real morbidity of the influenza virus in CF, and its impact on new *P. aeruginosa* colonization, needs to be further investigated because all previous studies were mainly observational and did not achieve clear-cut conclusions [4, 6, 11, 13, 15].

The 2009 pandemic A/H1N1 provided a good opportunity to study the consequences of influenza in a large number of CF patients. Indeed, as all patients were treated according to a consensual therapeutic protocol based on early and systematic use of oseltamivir and antibiotics, the variability due to different managements was anticipated to be considerably reduced. Indeed all patients were instructed to go immediately after onset of symptoms to the CF center to begin oseltamivir and antibiotic treatment as early as possible [16]. We took advantage of this, and designed a prospective case–control study to assess the consequences of influenza A/H1N1 infection on CF lung disease and determinate the impact of this proactive therapeutic strategy on new broncho-pulmonary colonizations.

## Patients and methods

### Study design

MucoFlu (Clinical Trials.gov registration number: NCT01499914) was a prospective study initiated by INSERM (Institut National de la Santé et de la Recherche Médicale) and APHP (Assistance Publique des Hôpitaux de Paris) to study A/H1N1 infection in CF patients during the 2009 pandemy. This prospective case–control study included patients with CF followed in 14 CF centers in France and presenting for clinics or routine hospitalizations during the pandemic period.

Cases were CF patients with A/H1N1 infection, defined by the association of influenza-like illness and identification of pandemic virus A/California/7/2009 (A/H1N1v) strain in nasal secretions using Polymerase Chain Reaction. Influenza-like symptoms were temperature > 38 °C and at least one of the followings: cough, sore throat, rhinorrhea, nasal and/or bronchial obstruction, stiffness, asthenia, dyspnea, chest pain, or family contagion. They were enrolled during the period of pandemics where clinicians and patients were particularly aware for pandemic flu symptoms. Moreover, the patients or the parents of the child enrolled in the Muco-Flu study all received an information sheet about symptoms of flu and were instructed to go immediately after onset of symptoms to the CF centre to receive virological evaluation and begin adequate anti-infectious treatment, based on oseltamivir as early as possible after the onset of the symptoms and concomitant administration of antibiotics [16]. Enrollment of cases was as exhaustive as possible. Sputum colonization were monitored at least at 1, 3 and 12 months after the influenza infection by collecting sputum specimen or oropharyngeal throat swabs. All the respiratory function tests performed during the 1 year follow-up were collected, Transplanted patients were excluded. Controls were defined as patients enrolled in the MucoFlu study who did not experience any influenza-like illness during the 2009 pandemic nor at the enrollment visit. They were enrolled during routine clinics or hospitalizations. Around one third of the whole population followed in the CF centers (371 patients out of 1185) was enrolled. The reason for this discrepancy is that all the patients did not come for clinics during this period and others refused.

Written informed consent was obtained from each patient and parents or guardians of children below 18 years old. The protocol was conducted in accordance with the Declaration of Helsinki and French law for biomedical research and was approved by the Ile de France II Ethics Committee.

### Study endpoints

For each patient, case or control, demographics, mutation in the *CFTR* gene, respiratory function, sputum colonization and antibiotic use were recorded. For each case, symptoms and management of influenza were collected (treatment with oseltamivir or other neuraminidase inhibitor, antibiotic treatment, hospitalization, oxygen therapy or noninvasive ventilation initiation or modification).

Study patients were followed from August 2009 to January 2011. Cases were reviewed at months 1, 3 and 12 after the influenza episode. Each follow-up visit included assessment of new bronchopulmonary colonization, oral and intravenous antibiotic treatment history, oxygen therapy and noninvasive ventilation, and pulmonary function testing if available (forced expiratory volume in one second (FEV1) and Forced Vital Capacity (FVC)). Pulmonary exacerbation was defined according to the Fuchs criteria [17]. Controls were followed according to site standard of care. Three bacteriological samples during the year of follow-up and a respiratory function test at 1 year were required as a minimum.

Bacteriological chronic colonization was defined by identification of the same microorganism during at least 3 months in at least 3 samplings. New bacterial colonization was defined as the first isolation of a microorganism or its recurrence after at least 1 year of negative sputum culture.

### Statistical analysis

Analyses were carried out with R. Data were expressed as mean (+/-SD). The endpoints at inclusion and at different times of follow-up were compared in cases vs. controls using Chi-2 or Fisher’s exact test for qualitative variables and Wilcoxon test for continuous variables. For further comparison of change during the year following the pandemic in the pediatric patients, we matched 1 case with up to 2 controls, according to age. CF-specific tables were used in order to compare respiratory parameters of cases versus controls and assess the percentile according to age and sex [17]. Missing FEV1 and CVF data were replaced by multiple imputation based on Multivariate Imputation by Chained Equations in R. Comparisons were made using non parametric tests, with correction for multiple imputation. All tests were bilateral at the 0.05 significance level.

## Results

### Patient characteristics at inclusion

Forty-four cases of pandemic A/H1N1 influenza infection were identified from August 2009 to February 2010 in 14 French centers. Three hundred and seventy-one CF controls were included from November 2009 to February 2010 in 14 centers following 1185 patients. Those patients were enrolled during routine clinics.

The characteristics of patients at the beginning of the study are summarized in Table 1. There were significant differences between cases and controls for age (14.9 years (+/-11) for cases and 20.1 years (+/-13.2) for controls; *p* = 0.01). Thirty three out of the 44 cases were below 18 years of age, including 8 patients below 6 years, 9 patients between 6 and 10 years and 16 patients between 10 and 18 years. Bronchopulmonary colonization with *P. aeruginosa* was significantly less frequent among cases (34 %) than controls (53 %). Cases had also a higher mean FEV1 (79 % (+/-28) predicted for theoretical value for sex and age) than the controls (65 % (+/-25). However, as expected, considering the younger age of the cases, this difference disappeared when FEV1 was expressed as percentiles of CF-specific FEV1, established in the CF population for better comparison (52nd percentile for cases and 51th percentile for controls; *p* = 0.78) [18]. Sixty six percent of the cases had been vaccinated with 2009/H1N1v adjuvanted vaccine. History of immunization was confirmed in 108 of the 371 controls. For the remaining 263 cases we do not know whether the absence of report was due to absence of vaccination or missing data.

**Table 1:** Characteristics of the cases and controls the year before A/H1N1 2009 pandemic influenza

	Cases (*n* = 44)	Controls (*n* = 371)	*p*
Male sex, n (%)	26 (59)	186 (50)	0.26
Age, years, mean (SD)	14.9 (11.0)	20.1 (13.2)	0.01
Mutation *CFTR* ∆F508/∆F508, n (%)	19 (43)	150 (40)	0.41
Bronchopulmonary bacterial colonization, n (%)			
*S. aureus*	34 (77)	234 (63)	0.063
*P. aeruginosa*	15 (34)	195 (53)	0.021
FEV1, %, mean (SD)	79 (28)	65 (25)	0.005
FVC, %, mean (SD)	87 (23)	79 (21)	0.073
Cystic fibrosis-specific FEV1, percentiles, mean (SD)	52 (32)	51 (30)	0.78
Antibiotics, number of treatments, mean (SD)			
Oral	2.2 (1.9)	2.5 (1.9)	0.17
Intravenous	0.8 (1.5)	1.1 (1.7)	0.20
Oxygen therapy, n (%)	2 (5)	3(4)	0.86
Noninvasive ventilation, n (%)	1 (2)	1(1)	0.69

Both are compared to reference data and expressed as percent predicted values, based on age, gender, and height

*FEV1* forced expiratory volume in one second, *FVC* forced vital capacity, *SD* standard deviation

### Characteristics of the influenza episode

During the pandemic A/H1N1 influenza episode, all cases had fever, 74 % experienced pulmonary exacerbation, 53 % myalgia and 59 % asthenia. Ninety-two percent received antibiotics (100 % of the adults and 89 % of the children, *p* = 0.56), including intravenous antibiotics for 21 %,. Thirty three percent were hospitalized (27 % of the adults and 35 % of the children, *p* = 0.72). Eighty-two percent of patients were treated with oseltamivir within the first 24 h after the onset of symptoms (55 % of adults and 90 % of children, *p* = 0.02).

Two adults had unfavorable outcomes after the influenza episode versus none in controls. The first case was a 39-year woman carrying the F508del/R347L mutations colonized with *P. aeruginosa.* Before the influenza episode, her FEV1 and FVC predicted were 28 and 40 % respectively. The patient received oseltamivir 48 h after the onset of symptoms. During the influenza episode, she experienced hypoxemia and hypercapnia with increase in oxygen therapy which led to initiation of noninvasive ventilation 1 month later. Lung transplantation was performed 5 months after the A/H1N1 influenza episode because of respiratory failure.

The other case was a 33-year F508del homozygous male patient. He was colonized with *Burkholderia cepacia* since 1987. His FEV1 predicted was 32 % before the influenza episode. The patient did not receive oseltamivir due to a delayed diagnosis. The respiratory condition of the patient experienced a severe deterioration during the influenza infection. Oxygen therapy was initiated during the influenza episode, and noninvasive ventilation 1 month later. Lung transplantation was performed 3 months after the influenza episode. One month after the transplantation, he died due to *B. cepacia* sepsis.

### Changes in bronchial colonization and respiratory function after the influenza episode

There was no significant change in FEV1 at months 1, 3 and 12 after the influenza episode (Table 2). Unexpectedly, there was even a systematic trend towards an increase in respiratory function 1 month after the viral episode. This may reflect the proactive symptomatic and anti-infectious treatment as indirectly suggested by the fact that cases received a significantly higher number of antibiotic treatments during the year after the influenza infection than controls (mean of 2.8 (+/-2.4) in cases versus. 1.8 (+/-2.1) in controls; *p* = 0.002). There was no difference for any new sputum colonization including *P. aeruginosa* after the influenza episode in cases compared to controls (30 % versus. 41 %, respectively; *p* = 0.60).

**Table 2:** Characteristics of cases and controls during the year after A/H1N1 2009 pandemic influenza

	n	Cases (*n* = 44)	n	Controls (*n* = 371)	*p*
Change in FEV1, %, mean (SD)					
Month 1	12	8.3 (19.2)	198	−2.3 (8.4)	0.08
Month 3	17	0.6 (7.8)	211	−2.5 (8.7)	0.14
Month 12	28	1.2 (13.3)	249	−1.8 (11.0)	0.67
Change in FVC, %, mean (SD)					
Month 1	12	7.4 (18.1)	198	−3.9 (8.8)	0.05
Month 3	17	0.7 (8.7)	212	−2.5 (8.9)	0.16
Month 12	28	1.9 (12.7)	252	−2.6 (9.8)	0.10
Antibiotics, number of treatments, mean (SD)					
Oral	44	2.8 (2.4)	371	1.8 (2.1)	0.002
Intravenous	44	0.6 (1.3)	371	0.8 (1.54)	0.42
New bronchopulmonary colonization, n (%)					
Any germ	44	13 (30)	371	151 (41)	0.60
*H. influenza*	44	1 (2)	371	32 (8)	0.13
*S. aureus*	44	3 (7)	371	39 (11)	0.42
*P. aeruginosa*	44	6 (14)	371	36 (10)	0.48
*B. cepacia*	44	0	371	5 (1)	0.42
*A. fumigatus*	44	3 (7)	371	39 (11)	0.42
Oxygen therapy, n (%)	44	2 (5)	371	30 (8)	0.41
Noninvasive ventilation, n (%)	44	2 (5)	371	12 (3)	0.65

Both are compared to reference data and expressed as percent predicted values, based on age, gender, and height

*FEV1* forced expiratory volume in one second, *FVC* forced vital capacity, *SD* standard deviation

As expected, the pediatric (<18 years) versus the adult cases did not differ significantly neither for FEV1, FVC changes or the number of antibiotic courses during the year after the influenza episode (Table 3). Because of many missing data for FEV1 and FVC, comparison were made using non parametric tests, with correction for multiple imputation. Average of data after imputation were very similar to those of observed data, with, as expected, larger standard deviations (data not shown).

**Table 3:** Characteristics of the cases during the year after A/H1N1 2009 pandemic influenza according to age

	n	Cases <18 years (*n* = 33)	n	Cases ≥18 years (*n* = 11)	*p*
Change in FEV_1_, %, mean (SD)^a^					
Month 1	33	8.8 (18.0)	11	4.5 (16.92)	0.66
Month 3	33	0.8 (8.3)	6	0.5 (5.2)	0.97
Month 12	33	−1.5 (11.0)	9	3.9 (13.6)	0.26
Change in FVC, %, mean (SD)^a^					
Month 1	33	5.4 (15.3)	11	4.3 (12.2)	0.88
Month 3	33	0.9 (8.1)	11	−0.8 (5.4)	0.60
Month 12	33	−0.6 (10.9)	11	6.4 (13.9)	0.11
Antibiotics, number of treatments, mean (SD)					
Oral	33	2.7 (2.2)	11	3.3 (2.8)	0.57
Intravenous	33	0.5 (1.1)	11	0.9 (1.6)	0.34
New bronchopulmonary germ colonization, n (%)					
Any germ	33	13 (39)	11	0	0.013
*H. Influenza*	33	1 (3)	11	0	0.54
*S. Aureus*	33	3 (9)	11	0	0.29
*P. Aeruginosa*	33	6 (18)	11	0	0.12
*B. Cepacia*	33	0 (0)	11	0	1.00
*A. Fumigatus*	33	3 (9)	11	0	0.32

Both are compared to reference data and expressed as percent predicted values, based on age, gender, and height

*FEV*_*1*_ forced expiratory volume in one second, *FVC* forced vital capacity, *SD* standard deviation

^a^Missing data were imputed

As a whole, children infected with A/H1N1 influenza experienced significantly more frequently a new bronchopulmonary bacterial colonization in comparison to adults (39 % versus 0 %; *p* = 0.013), although the level of significance was not reached for any microorganism (*P. aeruginosa*, *Staphylococcus aureus, Haemophilus influenzae, B. cepacia and Aspergillus fumigatus*). Interestingly however, the largest difference in incidence was for *P. aeruginosa* (6 new colonizations in the pediatric population versus none in the adults, *p* = 0.12).

As controls were very different from cases for several parameters as age, bacterial colonization and respiratory function, we compared pediatric cases and age–matched controls in order to detect whether the increased incidence in *P. aeruginosa* new colonization was related to the influenza infection or simply to the difference in age (Table 4). Age and FEV1 were similar at baseline in this matched subset. The increase for new *P. aeruginosa* colonization among the patients who experienced influenza infection versus the controls disappeared. The overall incidence of new colonization was even somewhat larger in controls, especially due to more new colonizations with *H. influenzae* in the controls in comparison with cases (16 % versus 3 %; *p* = 0.04). Controls displayed a trend to more oral antibiotic courses than controls during the year of follow-up.

**Table 4:** New broncho-pulmonary colonization and antibiotic courses of pediatric cases and age-matched controls during the year after A/H1N1 2009 pandemic influenza

	n	Cases (*n* = 33)	n	Controls (*n* = 55)	*p*
Age	33	9.7+/- 4.7	55	9.5+/- 4.8	0,9
Antibiotics, number of treatments, mean (SD)					
Oral	33	2.7 (2.2)	55	2.4 (2.6)	0.40
Intravenous	33	0.52 (1.12)	55	0.56 (2.01)	0.33
New bronchopulmonary colonization, n (%)					
Any germ	33	13 (39)	55	26 (47)	0.47
*H. influenza*	33	1 (3)	55	9 (16)	0.04
*S. aureus*	33	3 (9)	55	6 (11)	0.78
*P. aeruginosa*	33	6 (18)	55	6 (11)	0.44
*B. cepacia*	33	0	55	1 (2)	0.40
*A. fumigatus*	33	3 (9)	55	4 (7)	0.94
Oxygen therapy, n (%)	33	0 (0)	55	0 (0)	1
Noninvasive ventilation, n (%)	33	0 (0)	55	0 (0)	1

Both are compared to reference data and expressed as percent predicted values, based on age, gender, and height

*FEV1* forced expiratory volume in one second, *FVC* forced vital capacity, *SD* standard deviation

## Discussion

This study is the first to prospectively assess the impact of an influenza episode and of a proactive anti-infectious strategy in a large case–control cohort of patients with CF. Results clearly show that pandemic A/H1N1 influenza did not lead to significant increase in new colonization nor changes in respiratory function during the year after the influenza episode. Early antiviral therapy seems crucial to limit deleterious consequences of A/H1N1 disease in CF patients.

### Mitigation of potential study bias

We deliberately chose to evaluate only the patients with virologically proven influenza infection. As we did not perform influenza serology in the controls enrolled in the study, we do not know if any of the controls may have contracted asymptomatic H1N1 before the start of recruitment and during the study period. To minimize this bias, the controls were patients who never experienced any symptoms of influenza infection during the pandemic period.

Clearly, controls are very different from cases for age, history and clinical parameters, which precludes to draw firm conclusions from the comparison of the 2 populations. To address this main drawback, we first checked from the french CF 2010 registry that the controls are representative of the general CF population. We then performed age-matched comparison in the pediatric patients, one case matched with up to 2 controls according to age. Age, FEV1 and bacteriological pattern of colonization were similar at baseline, demonstrating the comparability of this matched subset. Two different reasons may explain the majority of children among the infected cases. The first one is that the 2009 influenza A (H1N1) pandemic predominantly affected young age groups [19]. In France, according to the French influenza network, the less than 15 years of age were the most frequently infected, mainly the 5–9 years, as assessed from out-patients visits for Influenza like Illness [20]. The second one may be related to the increased awareness for clinical symptoms of the parents of CF children and the pediatrician, based on reports of severe outcomes in children [19].

A large proportion of lung function data are missing. This is because on the one hand, clinicians provided data according to local follow-up, and on the other hand, young children did not perform reliable respiratory functions. To handle missing data and minimize this problem, we used multiple imputation model and checked that the average of data after imputation were very similar to those of observed data, Nevertheless, as a substantial proportion of lung function data is missing at 1 month, it is impossible to draw any meaningful conclusions on the short term effect of influenza infection. Mid -term effects of an influenza episode can be better estimated, as 2/3 of patients contributed data.

Two third of the cases were vaccinated with 2009/H1N1v adjuvanted vaccine but nevertheless underwent influenza infection. This is in line with our previous study, where we demonstrated that patients with CF seroconverted less than the general population [21]. As we miss information about pandemic H1N1 immunization in 2/3 of the controls, it is impossible to draw any firm conclusion about the influence of pandemic (H1N1) vaccination in our cohort.

### Benign short term evolution of A/H1N1 influenza infection

Although 75 % of patients experienced pulmonary exacerbation during the pandemic A/H1N1 influenza episode, the majority of patients had a benign evolution after infection with A/H1N1 influenza. This result may be partly related to the fact that 75 % of cases were children with moderate disease. Two patients out of 44 had severe decompensation of their respiratory disease, leading to lung transplantation in the months after the influenza episode. Those patients were the most severe of the sample (respective FEV1 predicted at 28 and 32 %, versus 82 % (36–138 %) for the 42 remaining others). Infection with *B. cepacia* in one of the 2 patients is possibly a risk factor of worse evolution after A/H1N1 infection as suggested by the fact that 8 controls also infected with *B. cepacia* did not have any modification of their respiratory function during the study period.

Those observations are in line with previous studies of A/H1N1 influenza which showed that the majority of patients were below 18 years of age and had a benign short term evolution both in CF [22–25] and outside CF [26–28]. Respiratory worsening including mechanical ventilation and premature death has been reported only in a small number of patients, mainly in adults with severe CF lung disease [15, 22]. In the retrospective study of Viviani et al [15], H1N1 infection had a significant impact on the disease course in patients with CF (110 patients diagnosed with A/H1N1 infection): 53 % required intravenous antibiotic therapy, 48 % were hospitalized for an average of 12.9 days and 31 % required oxygen treatment during the time of infection. Six patients, all with severe lung disease required intensive care unit, 5 of them needing mechanical ventilation and 3 died during the course of the infection. Although the authors state that, as a whole, FEV1 did not change after A/H1N1 infection, this parameter was significantly lower in hospitalized patients. However the absence of case control design in both of these studies does not allow any formal conclusion [15, 22].

### Maximal early treatment is mandatory after A/H1N1 infection

Systematic early prophylactic treatment with oseltamivir within 48 h was recommended in CF patients by French Health Authorities in case of influenza-like symptoms in this pandemic context, taking into account the potential bad prognosis. In our study, 82 % of the cases were treated with oseltamivir, 92 % with antibiotics and one third were hospitalized. Although not achieving statistical significance, a transient improvement of the respiratory function was observed at month 1, most probably due to this increased proactive treatment. This could also explain why the proportion of new colonization with *H. influenza* in patients < 18 years was higher in controls compared to cases as this bacteria may have been eradicated by antibiotic treatment prescribed during the flu episode.

Very importantly, in the two most severe cases, oseltamivir was either not administered or the delay of administration was more than 48 h after the beginning of the episode. On the opposite, all other benign cases received oseltamivir within 24 h after the beginning of the symptoms. A recent study also showed that early treatment with oseltamivir in hospitalized children decreased the clinical severity of A/H1N1 influenza [29]. Conversely, late treatment with oseltamivir treatment (>48 h after onset of symptoms) was shown to be associated with more severe exacerbations [23]. We point out however, that the absence of randomized controlled data in our study does not allow to draw any firm conclusion about specific benefit of neuraminidase inhibitors in CF. Nevertheless, our results suggest that early management and follow-up is crucial to limit deleterious consequences of A/H1N1 disease in CF patients.

### Case–control study shows a limited impact on lung disease in the year after A/H1N1influenza infection

During the year following A/H1N1 infection, there was no significant increase in oxygen therapy and noninvasive ventilation. We did not observe any significant changes in functional respiratory parameters (FEV1, FVC). These results are in line with other reports that also did not evidence any decrease of respiratory functions after A/H1N1 infection [15, 22, 24]. This may be related to a more close follow-up and increased proactive treatment as suggested by the fact that cases received a mean number of antibiotic treatments significantly higher during the year after the influenza infection in 2009 (mean of 2.8 +/- 2.4 in cases versus 1.8 +/- 2.1 in controls; *p* = 0.002). Interestingly, antibiotic courses were not prescribed in the months following the influenza wave but rather all throughout the year. Although this increased “vigilance” is only a speculation, it must be underlined that the CF medical community was very concerned by the potential bad prognosis after A/H1N1 infection [19].

Chronic colonization with *P. aeruginosa* is one of the main prognosis factor in CF. In patients with CF, the increase in the number of *P. aeruginosa* colonization during winter has been strongly correlated with seasonal influenza [11], therefore suggesting that previous infection with influenza made the lung mucosa prone to bacterial colonization. Indeed, influenza viral infection can alter host defense equilibrium, by destruction and desquamation of the mucous membrane, increase in airway resistance and mucus production. This impedes clearance of inhaled microorganisms, hence promoting colonizing bacterial overgrowth and consecutive bacterial infections [30, 31]. This may on the one hand lead to the development of chronic *P. aeruginosa* infection in lungs hitherto uncolonised, or only intermittently colonised, and even initiate signs of chronic respiratory disease in children with CF. This is suggested in a prospective observational study, where 11 % of the cases initially free of *P. aeruginosa*, became colonized after A/H1N1 influenza infection [15]. On the other hand, virus may promote overgrowth, dispersion and invasion of chronic mucoid *P. aeuginosa* embedded in biofilm [32]. This is suggested by 2 observational studies showing a more frequent pulmonary deterioration following non-bacterial infection when chronic bacterial infection is present [33] and the increase in precipitins to *P. aeruginosa* coincidental with the viral infection [4].

We did not find such a difference in our study: 14 % of cases underwent a new *P. aeruginosa* colonization, but also 10 % of controls. Very importantly, the case–control design allowed correcting a possible bias related to age-dependant colonization. Indeed, the comparison of the pediatric cases versus the adult ones showed that patients below 18 years significantly underwent more new bacterial colonization during the year after influenza infection (39 %, for pediatric cases versus none for adults, *p* = 0.013) with a specific increase in *P. Aeruginosa* (6 pediatric cases versus none among the adults) (Table 3). After matching pediatric cases with controls according to age, this initial difference disappeared (Table 4). Similarly, a study in adults with CF did not find any change in sputum culture growth in the overall group before and after A/H1N1 infection [24]. This difference obtained by comparing pediatric with adult cases probably simply reflected the fact that children experienced more frequently new bacterial colonization because they were not yet colonized in contrast to adults. This also underscores the necessity of case–control studies for rigorous interpretation of data and avoiding potential bias of interpretation.

## Conclusion

The pandemic influenza A/H1N1 virus has now adopted the properties of a seasonal influenza virus in the post-pandemic period [34]. Therefore, our observations not only concern the specific pandemics of A/H1N1, but may be generalized to epidemics of seasonal A/H1N1 flu.

Our results suggest that a proactive antiviral and antibacterial early treatment may limit the impact of A/H1N1 in CF patients, quite particularly sputum colonization and lung disease progression. More generally, this underscores the need to treat early CF patients with respiratory viral infections and to prevent respiratory exacerbation with antibiotics.
